# Function and flexibility of object exploration in kea and New Caledonian crows

**DOI:** 10.1098/rsos.170652

**Published:** 2017-09-27

**Authors:** Megan L. Lambert, Martina Schiestl, Raoul Schwing, Alex H. Taylor, Gyula K. Gajdon, Katie E. Slocombe, Amanda M. Seed

**Affiliations:** 1 Department of Psychology, University of York, York, UK; 2 Department of Cognitive Science, Lund University, Lund, Sweden; 3 Haidlhof Research Station, University of Vienna and University of Veterinary Medicine, Vienna, Austria; 4 Department of Linguistic and Cultural Evolution, Max Planck Institute for the Science of Human History, Jena, Germany; 5 Comparative Cognition Unit, Messerli Research Institute, University of Veterinary Medicine Vienna, Medical University of Vienna, University of Vienna, Vienna, Austria; 6 Department of Psychology, University of Auckland, Auckland, New Zealand; 7 School of Psychology and Neuroscience, University of St Andrews, St Andrews, UK

**Keywords:** cognition, tool use, object play, corvid, parrot, object properties

## Abstract

A range of non-human animals frequently manipulate and explore objects in their environment, which may enable them to learn about physical properties and potentially form more abstract concepts of properties such as weight and rigidity. Whether animals can apply the information learned during their exploration to solve novel problems, however, and whether they actually change their exploratory behaviour to seek functional information about objects have not been fully explored. We allowed kea (*Nestor notabilis*) and New Caledonian crows (*Corvus moneduloides*) to explore sets of novel objects both before and after encountering a task in which some of the objects could function as tools. Following this, subjects were given test trials in which they could choose among the objects they had explored to solve a tool-use task. Several individuals from both species performed above chance on these test trials, and only did so after exploring the objects, compared with a control experiment with no prior exploration phase. These results suggest that selection of functional tools may be guided by information acquired during exploration. Neither kea nor crows changed the duration or quality of their exploration after learning that the objects had a functional relevance, suggesting that birds do not adjust their behaviour to explicitly seek this information.

## 1. Introduction

Unrewarded object exploration is widespread among non-human animals, and is thought to confer important benefits by allowing individuals to develop motor skills for foraging and engage in novel behaviour patterns that may lead to innovative foraging strategies [1–3]. Additionally, it might provide increased opportunities to learn about the affordances of objects and potentially develop more abstract concepts about ‘invisible’ physical properties such as weight and rigidity, which could provide a route to flexible problem solving and tool use [4]. The exploratory play of young humans is thought to scaffold such learning, whereby infants construct knowledge about an object's functionality through repeated interaction [5–7]. Consequently, unrewarded object exploration may serve as an important phylogenetic or ontogenetic precursor to tool use. Despite its potential importance in this domain, however, little is known as to whether any animals gain information about object properties through exploration, and whether any non-human animals can use exploration strategically to gain such information.

Some of the most pronounced object exploration is found among relatively large-brained birds and primates, with tool-using species generally exhibiting higher rates of object exploration than non-tool-using species [8–11]. For example, early comparative studies of 74 primate species found that tool-using *Cebus* [10] and great ape species [9,10] showed a broader repertoire of manipulations than non-tool-using primate species, or spent more time manipulating detached objects during development than closely related species which did not habitually use tools (chimpanzees vs. bonobos [12]). Among birds, some species of corvid and parrot, which readily explore and play with objects, demonstrate sophisticated problem-solving abilities in captivity as well as spontaneous and habitual tool use either in the wild (New Caledonian crows [13], see [14,15] for problem-solving experiments) or captivity (e.g. Goffin cockatoos [11,16]; greater vasa parrots [17]). Recent comparative studies within parrots and corvids have additionally shown that species proficient in tool use, either in the wild or in experimental contexts, showed high frequencies of combined object manipulation compared with non-tool-using species, either during ontogeny or in adulthood [18,19].

Despite this evidence, the nature of the link between object exploration and problem solving (including tool use) is relatively unexplored. What (if any) information is being gained from exploration of an object that may later translate to its functional use? Do animals change the way they explore objects in order to gain information about them? There is some evidence to suggest that tool-using primates such as chimpanzees and capuchin monkeys can learn about potential tools through exploration. For example, an early study found that chimpanzees were only able to solve a task involving a stick tool after they gained experience manipulating sticks in their home enclosure, suggesting that subjects learned something about the functionality of the sticks through exploration [20]. Additionally, great apes and capuchins perform significantly better at selecting functional (rigid) stick tools from a set of objects either after having manipulated the objects themselves or after watching an experimenter demonstrate the properties of the objects ([21,22], but see [23,24]). In a related study, non-tool-using marmosets also learned to select functional tools after gaining experience with similar tasks [25].

A related question involves whether exploration is used intentionally to seek information. Individuals might incidentally gain information about the objects' hidden structural properties as they are manipulated, but do they alter the quality or duration of their exploration to learn about these properties once they have been confronted with a task? This type of behaviour is evident in young children who increase their exploration in order to learn about causally ambiguous stimuli [26]. Similarly, when choosing among stone tools for nut-cracking, wild capuchin monkeys resort to techniques such as tapping or moving stones that do not possess reliable visual cues about weight as a functional property (e.g. when choosing between identical stones, or when larger stones are lighter than smaller stones [27]). By contrast, chimpanzees did not change their exploratory behaviour in order to seek explanations about why familiar objects suddenly functioned differently in the absence of visual cues (e.g. counterweighted blocks which no longer stood upright [28]).

The current literature, therefore, provides some evidence that primates such as chimpanzees and capuchin monkeys can learn about objects through exploration [21,22], and even change their exploratory behaviour to gain information about potential tools, though the degree to which this involves abstract representations of object properties such as weight and rigidity remains a matter for debate [29,30]). Our study aimed to determine whether (i) information about object properties is gained through exploration and can be applied in a problem-solving context; and (ii) if knowledge about the potential use of an object as a tool changes exploratory behaviour.

We presented kea, a highly explorative species that can use tools in captivity but does not use tools in the wild [31], and New Caledonian crows, proficient tool users and manufacturers in both wild and captive settings [15,32], with a series of experiments to investigate the function and flexibility of exploration in learning about structural object properties. The behavioural differences between these two species allowed us to test the potential role of exploration for a habitually tool-using species (i.e. whether tool-using birds are more likely to attend to object properties during manipulation or directly seek information), as well as how the kea's pronounced exploration into adulthood might function (i.e. what information they glean from their exploration and how flexible this behaviour might be). As both kea and New Caledonian crows excel at solving physical problems, examining their exploratory behaviour provides insight into how they actually acquire information to solve these tasks, as well as what object features they attend to.

In Experiment 1, we gave subjects the opportunity to explore sets of similar novel objects that varied in their underlying structure (i.e. identically sized blocks of different weights and identically sized rope pieces with different rigidity). These objects also varied visually in terms of colour and pattern, so each object was unique and one of these visual features varied systematically with the structural properties (e.g. all heavy blocks were striped). We investigated whether they gained information from their object exploration which could later be used to solve a task. They were trained to solve the task with different objects (a heavy ball that collapsed a platform, and a rigid stick that could be used to extract a reward from a pipe). Following this, subjects received forced-choice trials in which objects from the explore phase were presented to see if subjects could select the functional tool based on their previous experience with them. By presenting the objects in two phases, half before and half after the task was first introduced, we also aimed to investigate whether subjects changed the duration or quality of their exploration to gain information about the objects' properties once they had had the chance to learn that half of the objects would function as tools. In Experiment 2, we presented subjects with a new set of blocks, this time with colour as the visual feature corresponding with structure (rather than pattern), to determine whether poor performance with the blocks in Experiment 1 resulted from difficulties in discriminating between heavy and light objects, or in associating weight with pattern. Finally, each subject's performance in Experiments 1 and 2 was compared with test trials featuring a new set of objects that subjects did not have the opportunity to explore or interact with prior to testing (Experiment 3).

It was expected that birds which spent longer exploring objects or engaged in more functional behaviours (potentially able to reveal the hidden structural properties of the object; e.g. picking up a block to reveal weight versus touching the surface of a block with the beak) would perform better in the tool-use trials. If subjects' exploration was driven by information seeking, they were expected to increase the amount of time they spent exploring similar (but visually novel) objects and/or to qualitatively alter their exploratory behaviour after learning that the properties of the objects had a functional relevance. Finally, if exploration generally provided information about the underlying structure of the objects, it was predicted that subjects would perform significantly better in Experiments 1 or 2, in which they were allowed to explore the objects prior to using them as tools, than in Experiment 3 when they were not allowed to explore.

## 2. General methods

### 2.1. Subjects

#### 2.1.1. Kea

Subjects were eight captive-born kea (one adult female, six adult males and one sub-adult male) housed at the Haidlhof Research Station (University of Veterinary Medicine and University of Vienna) in Bad Vöslau, Austria. Testing took place in mornings and afternoons just after feedings. The birds had previously participated in numerous cognitive experiments. Three of the kea (KE, FR and PI) had participated in studies focusing on tool use and object exploration, some of which involved subjects using rigid stick tools (e.g. [19,31,33]). As we were interested primarily in changes in exploratory behaviour before and after facing the tool-use task, our baseline phase would have allowed us to consider any biases for such objects and measure how subjects' behaviour changes after encountering the tool-use task. Kea are omnivorous, generalist foragers that do not naturally use tools in the wild, although they generally forage under rocks or for embedded foods [34]; therefore, weight may be a particularly salient property for them.

#### 2.1.2. Crows

Subjects were six wild-caught New Caledonian crows (three juvenile males; one adult male and two adult females) housed in captivity for approximately five weeks prior to the start of testing. Age was determined from mouth coloration and sex was determined from body size [35]. Prior to testing, all birds had been trained to drop stones into a Perspex box, causing a platform to fall and release a reward. During testing the birds concurrently participated in a hand tracking study [36], but otherwise had no experience with artificial tasks that required them to attend to structural object properties, although we note that subjects likely had experience using stick tools in the wild. Consequently, rigidity may be a particularly salient structural property for the crow subjects.

### 2.2. Testing compartments

#### 2.2.1. Kea

For the kea, all object exploration trials (Phases 1 and 3) took place in the waiting compartment, while test trials (Phase 4) took place in the neighbouring testing compartment. The waiting and testing compartments were attached to the main aviary and featured opaque sliding doors so that subjects were in visual but not auditory isolation from conspecifics. The experimenter was visible throughout exploration and test trials, and during test trials was present in the same compartment as the subject but remained motionless with gaze fixed on the apparatus until the subject made a choice, at which point the experimenter removed the remaining object. The experimenter stood next to the entrance to the testing compartment, so that subjects walked into the compartment and immediately had their backs facing the experimenter as they selected an object.

#### 2.2.2. Crows

Both exploration and test trials took place in a designated testing aviary attached to each group's main aviary, such that each group had its own distinct testing compartment. The experimenter was never visible and remained on the outside of the testing compartment filming all interactions during object exploration trials. Throughout test trials, the experimenter only entered to re-bait the apparatus and begin a new trial.

## 3. Experiment 1: learning about weight and rigidity through exploration

**Figure 1.**
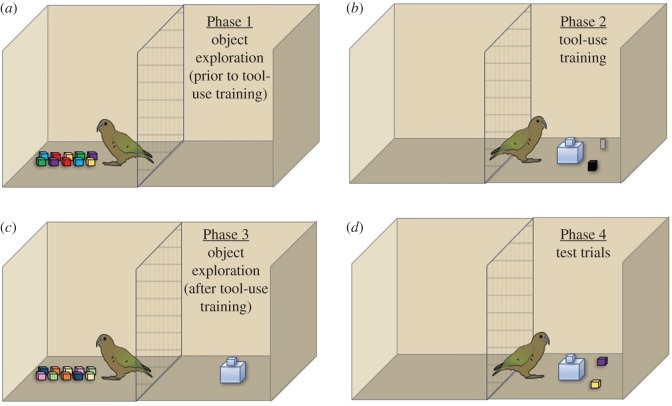
Overview of Experiment 1 testing phases, using the block set as an example.

Experiment 1 was aimed at determining whether exploration provided birds with information about the structural properties of objects that would enable them to subsequently choose a functional tool in a tool-use task, and whether subjects changed the quality or duration of their exploration to learn about these properties after they had experience that some of the objects could function as tools. All test phases are outlined in figure 1 for clarity and are described in further detail under Procedure.

### 3.1. Set-up and apparatus

Each species was tested with two different object sets, each of which focused on a distinct structural property (rope set: rigidity; block set: weight). Each set consisted of 20 similar objects (e.g. 20 lengths of rope, all identical in size and material) that varied in terms of their observable (colour, pattern) and unobservable properties (structure; in the case of the ropes, half had a stiff piece of wire inside). For each object set, one of the observable features (e.g. colour for the rope set) was relevant in that it corresponded with the structure of the object, while the alternative feature was irrelevant (e.g. pattern for the rope set). The presentation of object sets was counterbalanced such that some subjects began testing with the rope set while others began with the block set. The birds received two phases of exploration with these objects: 10 objects of the set were provided before they learned to solve a problem, and 10 afterwards.

#### 3.1.1. Blocks/weight

**Figure 2.**
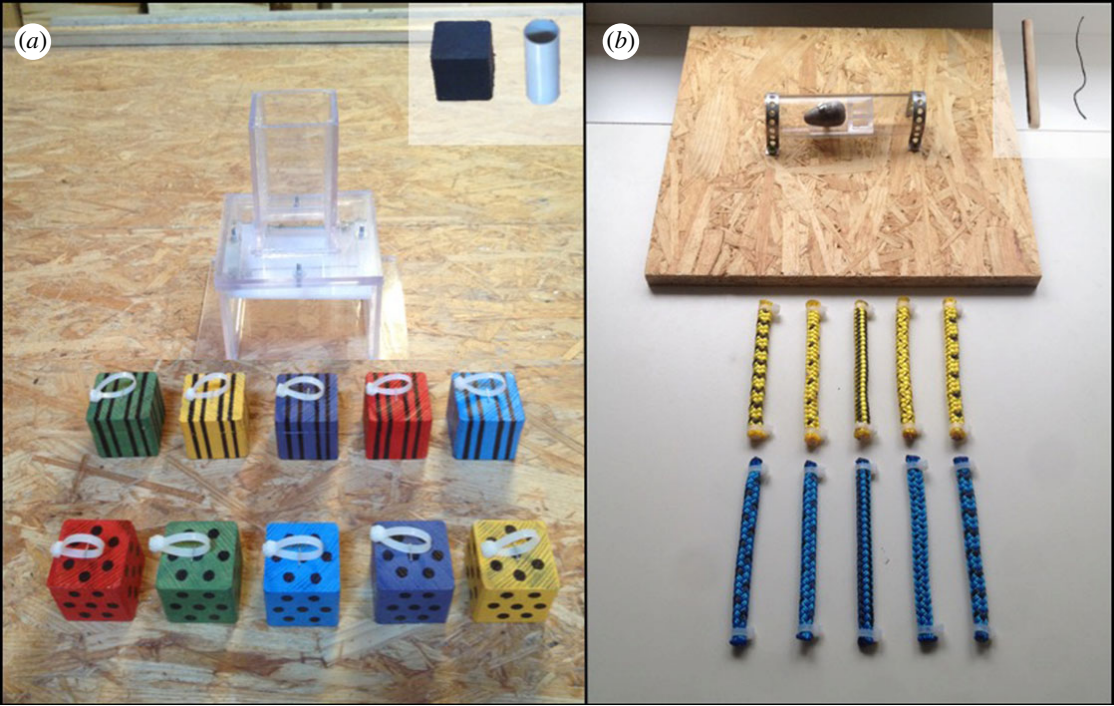
Example object sets and apparatuses used with the kea. (*a*) Block set: individuals must select a heavy block (weight corresponds with pattern) to insert into the opening of the box to collapse the platform inside. (*b*) Rope set: subjects must choose a rigid rope (rigidity corresponds with colour) to push out the weighted box in the middle of the tube. Training objects for each set are shown in the insets of each image.

The ‘weight’ object set for the kea consisted of 20 wooden blocks (4 × 4 × 4 cm), half of which were light (45 g) while the other half were heavy (125 g). All heavy blocks featured a striped pattern and all light blocks featured dots (figure 2*a*). Ten different colours were used within the set, with each colour featured on both a heavy and light block, thus making colour an irrelevant feature. Owing to size restrictions, the crows were presented with an analogous set of objects consisting of small clay balls (2 cm diameter), with the heavy balls weighing 44 g and the light balls weighing 0.04 g.

For the test trials (Phase 4, described below), the birds had to drop the blocks or balls into an opening in the top of a Perspex box (figure 2*a*) [37]. If the birds selected a heavy block, the weight of the block would cause a platform within the box to collapse, consequently releasing a reward (1/8 of a peanut for the kea, a similarly sized piece of meat for the crows) from an opening in the bottom of the box. In order to prevent mistrials, the platform was held in place by an electromagnet that was operated remotely by the experimenter, so that when subjects inserted the correct object, the experimenter pressed a button on a remote that was hidden behind a clipboard held by the experimenter throughout the session, causing the platform to collapse.

#### 3.1.2. Ropes/rigidity

The ‘rigidity’ object set consisted of 20 pieces of rope (13 cm long, 0.75 cm diameter), half of which had a piece of gardening wire inserted in the middle, thus allowing the rope to function as a stick tool, and the other half of which were flexible and non-functional (figure 2*b*). Note that wire added a minimal amount of weight (2 g) to the rigid rope. The relevant observable property for the ropes was colour, so that all of the functional ropes were yellow, for example, while the other half were blue. Pattern was an irrelevant feature, with one functional and non-functional object marked with identical patterns so that the object set featured 10 different patterns in total.

For the test trials, kea subjects had to select a functional rope with a wire inserted in order to push a reward out of a Perspex tube (3.5 cm diameter, 11 cm long) mounted to a wooden board (figure 2*b*). The reward was presented in a small Perspex box attached to a weight (115 g) to ensure that it could only be dislodged with the functional rope. Owing to species differences in foraging techniques, a separate apparatus was used with the crows so that subjects raked in, rather than pushed, their food from an artificial crevice (opening 1.5 cm × 11 cm). For both apparatuses the reward was always presented at the same distance from the opening across trials.

### 3.2. Procedure

#### 3.2.1. Phase 1. Object exploration (prior to training)

This stage served as a baseline measure of how subjects interacted with the objects prior to learning that the objects could be used as tools. Ten of the twenty objects (five functional, five non-functional) were presented in the compartment, either on the ground (kea; figure 1*a*) or a table (crows). Subjects were given 10 min to explore the objects, during which time the experimenter filmed all interactions. If subjects did not interact with the objects during their first session they then received habituation trials (see the electronic supplementary material for detailed procedure) before continuing with exploration sessions. Subjects moved on to Phase 2 after they explored at least three exemplars of a functional and non-functional object (six total), or after a maximum of four sessions, to avoid the objects becoming irrelevant.

#### 3.2.2. Phase 2. Tool-use training

##### 3.2.2.1. Weight

After their initial exploration sessions, subjects were then trained on a tool-using task in which they were required to choose between a functional, heavy object (a heavy solid-black block weighing 125 g, or solid-black ball weighing 44 g for the crows) over a non-functional, light object (a hollow, plastic grey pipe: 4 cm long, 2.5 cm diameter, weight: 4 g) to drop into the opening of the box to collapse the platform inside and retrieve a reward (training procedure detailed in the electronic supplementary material). For both object sets, we used training objects that differed in their relevant structural properties, but which were visually different, in order to allow subjects to quickly learn the training task and incidentally gain experience with the structural property required to solve the problem. For example, one training item was much heavier than the other (although the weights did not exactly match those of the testing objects), so that subjects gained experience that the heavier object was functional. Both objects were presented equidistant from the apparatus. Subjects completed tool-use training after choosing the heavy black block on five consecutive trials.

##### 3.2.2.2. Rigidity

Subjects were required to choose between a functional, wooden stick tool (13 cm long, 1 cm diameter) over a non-functional piece of string (22 cm long, 0.5 cm wide) to retrieve a reward from a clear, Perspex tube (kea) or artificial crevice (crows). This task proved difficult for some kea (AN, LI), who were unable to retrieve the reward after several sessions with the stick tool inserted in the tube. These subjects were instead trained using the Perspex box apparatus from the block set, but with a new Perspex tube (height: 5 cm, diameter: 5 cm) on the top as the opening. Subjects were required to insert one end of the stick tool into the tube so that it made contact with the platform, and then apply enough force on the distal end of the stick to collapse the platform. These subjects were subsequently tested in Phase 4 using the same box apparatus. Subjects completed tool-use training after choosing the rigid stick tool on five consecutive trials.

#### 3.2.3. Phase 3. Object exploration (after training)

This stage allowed us to measure any differences in exploration patterns after subjects had encountered a task in which objects similar to those they explored could be used as tools. After reaching training criterion, birds were presented with the remaining 10 objects (half functional, half non-functional), and were again given 10 min for exploration upon first contacting an object. Different objects from those presented in Phase 1 were used (e.g. new colours or patterns) to reduce the likelihood that subjects would lose interest in the objects. In this stage, the apparatus used during training was baited and presented in full view, but not accessible, while the subjects explored the objects to increase the likelihood that they would associate the objects with the problem-solving task (figure 1*c*). In order to standardize comparisons between the two phases, the number of sessions subjects were given in this phase matched the number of sessions they were given in the first phase, such that if subjects had two exploration sessions in Phase 1, they were given two exploration sessions in Phase 3.

#### 3.2.4. Phase 4. Test trials

To test whether birds had gained information about the properties of the objects they explored relevant to their functionality as tools, subjects were presented with one test session of 10 consecutive trials in which they could choose between a non-functional and a functional object from the set of objects they had previously encountered. Subjects were allowed one choice per trial, and a success was measured when subjects chose and used the functional tool. The set-up was identical to that used in the final stage of Phase 2, where the objects were presented equidistant from either side of the apparatus, with the side of the functional object pseudorandomized and counterbalanced across trials (figure 1*d*). A choice was counted as the first object that the subject touched, so subjects could not use manipulation during this phase to inform their choice.

#### 3.2.5. Behavioural data

All exploration sessions (Phases 1 and 3) were coded in The Observer XT. As subjects met criteria for interacting with the objects at different times and, therefore, had different amounts of total object exploration time, the resulting behavioural data were then converted into several proportional measures for subsequent analysis (i.e. the duration of exploration behaviours divided by the total exploration time; see electronic supplementary material for full ethogram). Our three primary variables of interest were (i) general exploratory behaviour or all behaviours directed at the objects, (ii) functional behaviours which could potentially provide information about the underlying structure of the objects (e.g. picking up a block) and (iii) exploration directed toward the different object types (i.e. exploration of functional versus non-functional objects).

#### 3.2.6. Analyses

All data were analysed in IBM SPSS Statistics v. 21, using two-tailed tests with the significance level set at *p* < 0.05. Exact *p*-values are reported for all tests [38]. For all generalized linear mixed models (GLMMs), the full model is reported [39,40]. Data are reported from eight kea (blocks: *n* = 7; ropes: *n* = 6) and six crows (blocks and ropes: *n* = 6). Eleven individuals participated in experiments for both object sets (six crows and five kea) and three kea participated in the experiment for only one object set. A list of each subject's participation in each experiment can be found in the electronic supplementary material, table S1.

##### 3.2.6.1. Do birds learn from their exploration?

To determine whether, as a group, subjects performed above chance on test trials following exploration, a one-sample Wilcoxon's signed-rank test was used. To determine whether performance on the test trials was predicted by individuals' duration or quality of exploration across all exploration sessions (Phases 1 and 3 grouped), and whether this differed between species, a binomial GLMM (GLMM1) with a logit-link function was run with the number of correct trials as the numerator and the total number of trials (*n* = 10 for each subject) as the denominator. Fixed factors entered into the model were (i) total proportion of trial time spent exploring the objects (pre- and post-exploration grouped), (ii) total proportion of exploration time spent engaging in functional behaviours (pre- and post-exploration grouped) and (iii) species. Each individual had one data point per set entered into the model, resulting in 25 data points. Subject and object set were entered as random factors to control for repeated sampling.

We then ran a separate binomial GLMM (GLMM2) with a logit-link function to examine, on a trial-by-trial basis, whether subjects performed better on trials that included objects they had previously chosen to explore with functional behaviours, and whether this covaried with species. Trial outcome (correct versus incorrect) was entered as the binary target variable into the model. Fixed factors entered into the model were (i) experience with the objects (no functional behaviour directed towards either object, functional behaviour directed towards the functional object, functional behaviour directed towards the non-functional object or functional behaviour directed towards both objects), (ii) species and (iii) the interaction between species and prior experience (*n* = 250; 10 data points per individual per set). Subject and object set were entered as random factors to control for repeated sampling.

##### 3.2.6.2. Do birds alter their exploration to gain information about objects?

To determine whether individuals changed their behaviour depending on condition (pre- or post-tool-use training) and whether this differed depending on species, three separate GLMMs (3–5) were run, each featuring condition (pre- or post-tool-use training) and species as fixed factors, and individual and object set as random factors. The target variables for each GLMM were (i) the proportion of trial time spent exploring all objects (GLMM3), (ii) the proportion of time spent exploring functional objects (GLMM4) and (iii) the proportion of trial time engaged in functional behaviours (see electronic supplementary material, table S2 for examples; GLMM5). Each individual had two data points per object set entered into each model (one for pre and one for post), resulting in 50 data points in total. All proportional data were transformed for normality using the arcsine square root transformation [41, p. 248], and all GLMMs were run using a normal probability distribution and identity link function. We note that the arcsine transformation may not be a preferred method of data transformation [42], and we therefore ran additional pairwise comparisons corroborating the results of GLMMs 3–5 which are detailed in the electronic supplementary material.

### 3.3. Results

#### 3.3.1. Performance on test trials and relationship to exploration

**Figure 3.**
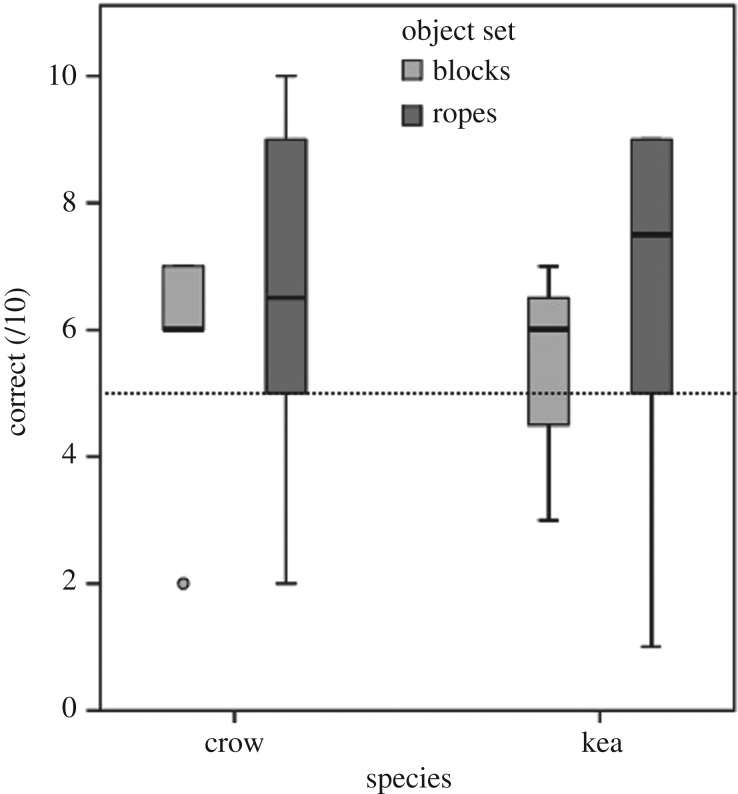
Boxplots illustrating the proportion of correct trials scored on either the block or rope set. Minimum and maximum values, median and 25th and 75th percentiles are shown. Dotted line denotes chance performance at 50%.

Each subject's performance for all experiments is detailed in the electronic supplementary material, table S4. Overall, four out of the 12 individuals that completed all four testing phases for the rope set (kea: one adult male and one sub-adult male; crows: two sub-adult males) performed above chance in the test trials set (9 or more of 10 trials correct, binomial test with chance probability of 0.5: *p* = 0.021), and no subjects (0/13) performed above chance for the block set. As a group, there was a trend for subjects' average test performance for both sets to be above chance (figure 3), but not significantly so (one-sample Wilcoxon's test: *p* = 0.074). A generalized linear mixed model (table 1, GLMM1) revealed that there were no significant between-species differences in performance on the test trials, and that performance on the test trials was not predicted by the total proportion of time that subjects spent exploring objects (Phases 1 and 3 combined), or the proportion of exploration time engaged in functional behaviours (Phases 1 and 3 combined).

**Table 1.** Results from Experiments 1–3. Full models are reported. Table includes dependent variable (DV), fixed effects, *F* statistic (*F*), degrees of freedom (d.f.) and coefficients ± standard error (s.e.). Random effects column includes the estimated variance component (EVC) for the random effect of subject, including standard error and significance. Significant effects (*p* < 0.05) are in italics. Note that ‘object set’ was included as a random factor but did not contribute to the model due to its small number of levels (i.e. removing it from the model had no influence on the results).

model/DV	fixed effects	*F*	d.f.1	d.f.2	coefficient ± s.e.	*p*-value	random effects (subject)
Experiment 1							
GLMM1. Performance	species	0.051	1	21	−0.145 ± 0.644	0.824	EVC = 0.554
	all exploration	0.103	1	21	0.894 ± 2.784	0.751	s.e. = 0.354
	functional exploration	1.181	1	21	−3.141 ± 2.890	0.289	*p* = 0.118
GLMM2. Correct/	species	0.004	1	242	0.067 ± 0.781	0.950	EVC = 0.522
incorrect (trial-by-trial)	experience w/objects	0.131	3	242	0 = −0.032 ± 0.601	0.942	s.e. = 0.329
					1 = 0.040 ± 0.510		*p* = 0.113
					2 = −0.441 ± 0.489		
	species×	0.767	3	242	Crow*0 = −0.383 ± 0.909	0.513	
	expw/objects				Crow*1 = −0.619 ± 0.934		
					Crow*2 = 0.607 ± 0.900		
GLMM3. Exploration time	*species*	*26*.*82*	*1*	*46*	*−0.455 ± 0.099*	*0.000*	EVC = 0.105
	pre or post	0.532	1	46	−0.058 ± 0.072	0.469	s.e. = 0.011
	species×	0.143	1	46	0.039 ± 0.104	0.707	*p* = 0.162
	PrePost						
GLMM4. Exploring	*species*	*4*.*571*	*1*	*46*	*−0.458 ± 0.173*	*0.038*	EVC = 0.041
functional objects	pre or post	0.192	1	46	−0.107 ± 0.130	0.663	s.e. = 0.029
	species×	2.491	1	46	0.296 ± 0.188	0.121	*p* = 0.156
	PrePost						
GLMM5. Functional	*species*	*21*.*608*	*1*	*46*	*−0.280 ± 0.083*	*0.000*	EVC = 0.007
exploration	pre or post	0.045	1	46	0.038 ± 0.068	0.833	s.e. = 0.007
	species×	0.321	1	46	−0.056 ± 0.099	0.574	*p* = 0.337
	PrePost						
Experiment 2							
GLMM6. Comparing	species	1.741	1	18	−1.207 ± 0.531	0.204	EVC = 0.308
performance Exps 1 & 2	experiment	0.193	1	18	−0.750 ± 0.393	0.666	s.e. = 0.253
	*species×*	*4*.*845*	*1*	*18*	*1.251 ± 0.568*	*0*.*041*	*p* = 0.224
	*experiment*						
GLMM7. Performance	species	0.096	1	7	−0.453 ± 1.460	0.766	EVC = 2.232
and exploration	exploration	1.424	1	7	3.421 ± 2.867	0.272	s.e. = 1.649
	functional exploration	1.347	1	7	1.924 ± 1.658	0.284	*p* = 0.176
Experiment 3							
GLMM8. Exploration	species	0.090	1	18	0.031 ± 0.646	0.768	EVC = 0.594
versus no exploration	*condition*	*12*.*209*	*1*	*18*	*−1.180 ± 0.410*	*0.003*	s.e. = 0.415
	species×	0.203	1	18	0.270 ± 0.598	0.658	*p* = 0.153
	condition						

When examining performance on each individual trial, prior experience with the exact objects used in that test trial did not predict whether subjects chose correctly on that trial (table 1, GLMM2). Similarly, there was no significant interaction between species and prior experience with the objects on whether subjects chose correctly in a given trial.

#### 3.3.2. Species differences in exploration

Each species' exploratory behaviour is summarized in table 2. There was a significant difference between species in the amount of time spent exploring the objects, with kea spending a significantly greater proportion of time interacting with the objects, engaging in functional behaviours and interacting with the functional objects relative to the non-functional objects than the crows (table 1, GLMMs 3–5).

**Table 2.** Summary of exploratory behaviour. Mean percentage of trial time (±s.e.) in each exploration phase that each species spent interacting with the objects (all exploration) or performing functional behaviours towards the objects (functional exploration), as well as mean percentage of exploration time spent interacting with functional objects.

	exploration phase		
behaviour category	pre	post	average of pre and post
kea (*n* = 8)			
all exploration	29 ± 8%	26 ± 5%	27 ± 6%
functional exploration	15 ± 3%	18 ± 5%	16 ± 4%
functional objects	53 ± 6%	43 ± 5%	48 ± 4%
crow (*n* = 6)			
all exploration	4 ± 2%	3 ± 2%	3 ± 2%
functional exploration	3 ± 2%	2 ± 1%	3 ± 2%
functional objects	20 ± 8%	32 ± 15%	26 ± 10%

#### 3.3.3. Pre- versus post-exploration

Each individual's exploratory behaviour for Phases 1 and 3 is summarized in the electronic supplementary material, table S3. There was no significant difference in the proportion of time that subjects spent exploring, engaging in functional behaviours or exploring functional objects before or after encountering the tool-use task; table 1, GLMMs 3–5), and these were also not predicted by an interaction between phase (pre- or post-tool-use training) and species (table 1).

## 4. Experiment 2: block set–colour versus pattern as a relevant feature

The results from Experiment 1 showed that four individuals performed significantly above chance in selecting functional objects to solve the task on the rope set, but not on the block set. This difference in performance could be linked to the materials themselves by reflecting a better understanding of rigidity, which unlike weight is visible following manipulation. Alternatively, it could be linked to the salience of the objects' visual features (e.g. colour relative to pattern). Colour, in particular, has been shown to be a highly salient stimulus in non-tool-using contexts, both in natural and in experimental settings [43–45]. If colour was a more salient feature associated with function, subjects were expected to perform better on this set of test trials relative to the block set in Experiment 1.

### 4.1. Procedure

Subjects were presented with a new block set using a similar procedure to Experiment 1, but this time with colour as the relevant feature associated with weight, and pattern as an irrelevant feature. Colours and patterns that the birds had not previously encountered in Experiment 1 were used. As the birds had already learned that the blocks could be used in a problem-solving task in Experiment 1, they received only post-training exploration sessions with the baited apparatus in full view. Each subject was given up to four sessions in which they were required to interact with three exemplars of each object type (functional and non-functional) before completing 10 test trials.

### 4.2. Analyses

To compare subjects' performance on test trials between Experiments 1 and 2, we ran a binomial GLMM with a logit-link function, featuring the proportion of correct trials as the target variable, and condition (colour versus pattern as the relevant feature) and species as binary fixed factors, as well as the interaction between species and condition. Subject identity was entered as a random factor. Each individual had one data point per object set entered into the model, resulting in 22 data points total in the model (*n* = 11 individuals).

We ran an additional binomial GLMM with a logit-link function to again determine whether, similar to Experiment 1, performance on the test trials was predicted by individuals' duration or quality of exploration, and whether this differed between species. The proportion of correct trials was entered as the target variable, and fixed factors entered into the model were (i) total proportion of trial time spent exploring the objects, (ii) total proportion of exploration time spent engaging in functional behaviours and (iii) species. Each individual had one data point entered into the model, resulting in 11 data points.

### 4.3. Results

While no subjects performed above chance for the weight set in Experiment 1, when the relevant feature was pattern, three kea (PA, PI and RO) and one crow (AZ) performed above chance when the relevant feature was switched to colour. Two of these individuals (PA and AZ) had also performed above chance for the rope set. Nonetheless, at a group level, performance was not significantly better when colour rather than pattern was the relevant feature on the blocks (table 1, GLMM6). There was no significant effect of species, however the interaction between species and condition was significant, with kea performing better than the crows when the relevant feature on the blocks was colour rather than pattern.

There was no significant difference between the species' performance on the test trials (table 1, GLMM7), and, similar to the results of Experiment 1, test performance was not predicted by the total proportion of time that subjects spent exploring, or the proportion of exploration time engaged in functional behaviours.

## 5. Experiment 3: no exploration control

### 5.1. Procedure

The results of Experiments 1 and 2 show that some kea and crows selected functional objects significantly above chance levels, and particularly when colour was associated with functionality. To determine whether these individuals are succeeding due to information gained during exploration it is important to test an alternative explanation, which is that subjects may be able to rapidly learn the reward contingencies in the test trials. To measure how well subjects would perform on test trials without any prior exploration of the objects, subjects were presented with a novel object set with novel relevant (colour) and irrelevant (pattern) features and were presented with 10 test trials in which they could choose between an unfamiliar functional and non-functional object to solve the task. We matched the control set to the exploration set on which the greatest number of individuals within each species performed significantly above chance. For the kea, this was the block set from Experiment 2 (3/6 individuals significantly above chance; colour-relevant feature) and for the crows, this was the rope set from Experiment 1 (2/6 individuals above chance; colour-relevant feature).

If exploration facilitated learning about the objects' functionality and thus enabled subjects to perform well on test trials in Experiments 1 and 2, it is expected that subjects that performed significantly above chance on test trials following exploration would perform worse in this experiment. By contrast, if subjects performed above chance in this condition as well as previous test trials, this would suggest that either (i) subjects may have generalized the rule that they learned during their exploration and applied it to these trials (e.g. colour correlates with functionality) or (ii) are able to rapidly associate success with the visual properties of the objects. Overall, it was predicted that if exploration enabled learning about object structure, performance of the group should be better on test trials following exploration than no exploration.

### 5.2. Analysis

To compare subjects' performance on test trials following exploration or no-exploration, a GLMM with a binomial probability distribution and a logit-link function was run featuring the proportion of correct trials as the target variable, condition (exploration versus no exploration), species and the interaction between condition and species as binary fixed factors, and subject as a random factor. Each individual had one data point per object set entered into the model, resulting in 22 data points total in the model (*n* = 11 individuals).

### 5.3. Results

#### 5.3.1. Do birds perform better after exploration versus no exploration?

**Figure 4.**
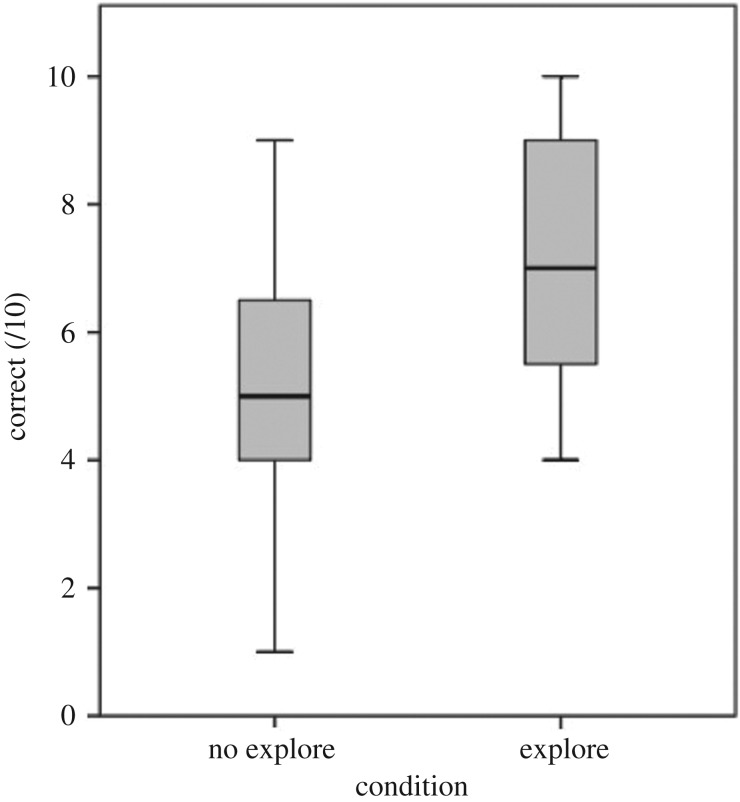
Boxplots illustrating the kea and crows' performance in the no-exploration and exploration conditions. Minimum and maximum values, median and 25th and 75th percentiles are shown.

All five subjects that performed significantly above chance after exploring the objects did not do so in this experiment (table 3). One subject which did not perform significantly above chance in any test sessions in Experiments 1 and 2 did so for this experiment. Performance on test trials was significantly predicted by condition, with subjects showing higher performance in conditions in which they could explore the objects beforehand compared to the no-exploration control condition (table 1, GLMM8). There was no significant between-species difference in scores for either condition or interaction between species and condition (figure 4).

**Table 3.** Individual scores for test trials after subjects were allowed to explore the objects (Experiment 2 for kea and Experiment 1 ropes for crow) or not (no exploration column). Italic denotes above chance performance.

		proportion of correct trials	
species	subject	exploration	no exploration
kea	PA	*1*.*00*	0.80
	PI	*0*.*90*	0.20
	FR	0.60	0.10
	LI	0.40	0.50
	RO	*0*.*90*	0.80
	JN	0.50	0.40
crow	BL	0.50	0.50
	AZ	*0*.*90*	0.40
	JO	0.70	*0*.*90*
	AT	*1*.*00*	0.40
	EM	0.60	0.50
mean		0.73	0.50
s.d.		0.21	0.24

## 6. General discussion

Overall, six birds performed significantly above chance on test trials in Experiments 1 and 2 when they could explore the objects beforehand (two for the rope set in Experiment 1, two for Experiment 2 and two for both). Importantly, in Experiment 3 all of these individuals performed at chance levels when similar visual cues were available but they had no opportunity to explore the objects. Five of these individuals encountered a no-exploration task that was directly comparable to the one in which they performed significantly above chance with exploration, providing support for the idea that exploration facilitated learning about the structural properties of the objects that informed test performance. One individual (FR) was significantly above chance for the rope set, but not above chance levels for the control block set. Although colour remained the functional feature in both of these object sets, the structural properties of the two object sets were different, so his data, although suggestive, cannot be interpreted in a comparable way to the other five birds.

It is also notable that one individual performed significantly above chance in Experiment 3, and therefore may have learned which objects were functional within the 10 trials without any exploration. It is uncertain whether this individual demonstrated rapid contingency learning in the absence of any understanding of functionality, or whether he may have generalized a rule (e.g. colour correlates with functionality) learned during his exploration and 20 previous colour-relevant test trials and applied it to these trials. It is also possible that some birds may have succeeded through adherence to a bias for a certain visual feature (e.g. colour) with little understanding of the task. For example, at least one bird (PI) showed a strong colour bias in the rope exploration condition that was resistant to contingency learning in the 10 test trials, with the bird choosing the incorrect object in 9/10 trials. Nonetheless, a comparison of all subjects' performance on exploration compared with no-exploration test trials revealed a significant effect of condition, with birds performing better in test sessions when they had previously explored the objects. That subjects only performed above chance when structure was associated with colour suggests that in the absence of causally relevant cues such as size or shape, colour, which has a high degree of ecological relevance, may serve as a more salient feature than pattern for forming associations between structure and visual features. It is important to note, however, that these results are not consistent at an individual level; for example, not all subjects that performed above chance with the rope set performed above chance with the block set, and vice versa, even when colour was the relevant feature in both cases. This is perhaps unsurprising given that the tool-use tasks were different for the two types of objects and required subjects to attend to different structural properties. It may be that some subjects excelled at attending to particular structural properties over others.

We were interested in initial performance on the task as a result of exploration rather than learning over trials, and therefore limited our number of trials to ten. With just 10 trials the performances that we predict (i.e. chance, or 5/10 on no exploration trials and above chance or minimum 9/10 on exploration trials) would not yield a significant difference at an individual level. While additional trials would have allowed for this, they would also have increased the chances of individuals learning during testing, potentially masking the effect of interest: prior exploration. At present, our findings are constrained by our limited sample size and therefore warrant follow-up with a larger number of individuals, and should be replicated over multiple object sets focusing on the same properties to further test the repeatability of individual performance and to rule out the possibility that individuals succeed merely by chance.

Although exploration may play a role in the ability to choose correctly between a functional and non-functional tool, it remains unclear what aspects of exploration drive this learning. In Experiment 1, performance in the problem-solving task was not predicted by the quality or quantity of experience that individuals had with the objects. These results are comparable to those of Manrique *et al*. [21,22], which show that capuchins and great apes are more likely to choose functional tools to solve a task if they had previous experience with those objects, and that this performance was not predicted by the quality of information provided, in terms of exploring the objects themselves or watching demonstrations by an experimenter. This lack of relationship may not be particularly diagnostic, however, as one could in principle get all the necessary information from a couple of manipulations, and conversely glean nothing from playing with all of the objects. If individuals meet a certain threshold level of exploration (that happens not to differ from their baseline as revealed in Phase 1) then we would not predict a correlation between exploration and success. An experiment in which a certain type or quantity of manipulation is required to reveal the hidden information, and unlikely to be performed during baseline, would be needed for this measure to be more revealing.

Both kea and New Caledonian crows did not appear to change their exploratory tactics after encountering the tool-using task, suggesting that their exploration was not driven by an attempt to gain information about the objects. Specifically, there was no difference in the amount of time that subjects spent exploring the objects, the proportion of functional behaviours directed toward the objects or the type of objects (functional or non-functional) explored after encountering the tool-use task. This was the case despite the baited apparatus being in full view, and even after subjects had completed their first object sets and had learned that the objects they were presented with could later be used as tools during the test trials. These results are consistent with those of Povinelli & Dunphy-Lelii [28], which suggest that chimpanzees do not engage in exploration in order to gain information about objects, but rather that exploration is driven by visual novelty. However, as above, it is possible that the birds' motivation to explore did differ between phases, but that we were not able to see evidence for this in the exploratory behaviour. Alternatively, the exploratory behaviour of these birds in the first phase may have already provided the necessary information about structure.

If the birds are not exploring objects explicitly to learn about their properties, and not all birds seem to learn from their exploration, this raises questions about each species' interaction with objects in the wild. Specifically, how do New Caledonian crows become such proficient tool users, and why are kea so explorative? One possibility, which warrants follow-up, may be that exploration is particularly important during development. The sample of 14 birds included four sub-adults (one kea and three crows). Although the limited number of sub-adults precludes statistical comparisons, these birds showed, on average, higher rates of exploration than their species average. Additionally, of the four birds that performed above chance in test trials for Experiment 1, three of these individuals were sub-adults. This suggests that perhaps there is a critical developmental stage during which individuals attend to and learn from stimuli in their environment, particularly when the costs of doing so are lower than in adulthood. The kea's readiness to interact with and manipulate new objects, even throughout adulthood, may additionally benefit these generalist feeders in discovering new food sources. Studies benefitting from a greater sample size might also consider intraspecific differences in task performance to determine the relative salience of particular structural properties in relation to species-typical foraging behaviour.

In terms of ultimate function, these results support the hypothesis that unrewarded object exploration provides information about object properties or affordances which can then be used to solve problems, but do not speak to other potentially overlapping functions of exploration such as honing manual skills or generating novel behavioural sequences [1]. Given its apparent costliness it is likely that exploration confers myriad benefits [46]. Further research explicitly addressing each of these benefits and their relative importance is needed to gain a richer view about how different animals learn about and interact with their environments in the absence of reinforcement (see [47] for a discussion).

In summary, this study suggests that kea and New Caledonian crow subjects may apply information generated from their exploration of novel objects to select functional tools in a later problem-solving task; however, we have no evidence that that they engage in strategic exploration to gain information about the functional properties of objects with respect to a problem-solving task. Although further data are needed to test other alternative (but not mutually exclusive) hypotheses, these data tentatively support the hypothesis that a key function of exploration in these birds is to provide opportunities to learn about object properties.

## Supplementary Material

Supplementary Methods and Results: Function and Flexibility of Object Exploration in Kea and New Caledonian Crows

## Data accessibility

Our data are deposited at: http://dx.doi.org/10.5061/dryad.dq04j [48].
