# Supplementary material for: Function and flexibility of object exploration in kea and New Caledonian crows
Source: R Soc Open Sci. 2017 Sep 27;4(9):170652. doi: 10.1098/rsos.170652 (PMC5627108; doi:10.1098/rsos.170652)
Supplement: Supplementary Methods and Results: Function and Flexibility of Object Exploration in Kea and New Caledonian Crows [file rsos170652supp1.docx]

**Electronic Supplementary Material: Function and flexibility of object exploration in kea and New Caledonian crows**

Megan L. Lambert, Martina Schiestl, Raoul Schwing, Alex H. Taylor, Gyula K. Gajdon, Katie E. Slocombe & Amanda M. Seed

**Subjects**

**Table S1.** Subject participation in each experiment. One additional crow did not interact with the rope set in Phase 1 and failed to complete training in Phase 2, and was excluded from the experiment. KE and ST dropped out of the study due to lack of motivation on the training trials (Phase 2) of Experiment 2 and AN dropped out due to lack of motivation on the block set for Experiment 1, and was not tested further. JN and RO were excluded from the rope set in Experiment 1 to ensure that they remained naïve to stick tool use for a future study.

| Species | Subject | Exp 1 | | Exp 2 | Exp 3 |
| --- | --- | --- | --- | --- | --- |
|  |  | Blocks | Ropes |  |  |
| Kea | PI | X | X | X | X |
|  | PA | X | X | X | X |
|  | FR | X | X | X | X |
|  | KE | X | X |  |  |
|  | LI | X | X | X | X |
|  | AN |  | X |  |  |
|  | JN | X |  | X | X |
|  | RO | X |  | X | X |
| Crow | BL | X | X | X | X |
|  | JO | X | X | X | X |
|  | ST | X | X |  |  |
|  | AT | X | X | X | X |
|  | EM | X | X | X | X |
|  | AZ | X | X | X | X |

**General Methods**

Subjects were presented with two sets of objects to explore, each of which focused on a distinct structural property (ropes: rigidity, blocks: weight). The relevant visual feature associated with rigidity for the rope set was colour (irrelevant: pattern), while the relevant feature associated with weight for the block set was pattern (irrelevant: colour). These two opposing features were included to make it more difficult for the birds to learn over the course of the test trials which objects were functional if they had not learned these contingencies during exploration. It also ensured that the objects used on each trial were visually unique so that birds would not continue to choose the same object they were rewarded for on previous trials, a potential confounding variable in other studies (discussed in Manrique *et al.* 2010; although note that stimulus generalization could still potentially occur, Honig & Urcuioli 1981).

**Experiment 1**

**Procedure**

***Phase 1. Object exploration (pre-training)***

Object exploration trials lasted up to thirty minutes, or until ten minutes after subjects first made physical contact with the objects. If subjects did not interact with any of the objects during the first ten minutes of the first object exploration session (as was the case for most of the crows), this procedure was paused and they were presented with several additional steps (Phase 1b) in order to first overcome potential neophobic responses while ensuring subjects did not over-habituate to the objects such that they would become an irrelevant feature of the birds’ environment.

#### ***Phase 1b. Habituation.***

For individuals that failed to interact with the objects during the first 10 minutes of encountering them, a habituation phase was introduced. This was needed for most of the crow subjects, but none of the kea. During these habituation sessions the objects were once more placed in the testing compartment but were now placed next to 10 small pieces of meat so that subjects had walk past and remain near the objects in order to retrieve all of the pieces. These individual habituation trials lasted 30 minutes for each bird. If subjects i) consumed all of the meat within one standard deviation of the time it took in baseline trials with only meat present and no objects (collected prior to the start of the experiment) and ii) didn’t show any startle behaviour (e.g., jumping jacks, described in Heinrich 1988) when approaching the objects, they began their first post-neophobia exploration session the following session. If subjects interacted with any of the objects by picking them up in the beak, a ten-minute timer was started from the moment of contact, and these ten minutes were considered the subject’s first post-neophobia exploration session.

If subjects still did not meet the criterion for retrieving the meat after three sessions, the objects were then presented in the subjects’ home aviary with meat nearby for up to three hours over two days, where all birds within that family group could see and potentially interact with the objects. If any birds interacted with the objects during this time this was counted toward that individual’s criterion for number of objects explored, and individual exploration sessions began the following day. If subjects still did not interact with objects during this time they were given a maximum of four more individual habituation sessions before being excluded from the study. No birds were excluded due to neophobia.

#### Phase 2. Tool use training.

***Weight.*** For the weight object set, subjects were trained to drop a heavy block into a Perspex tube. Subjects were trained in a step-wise manner, so that initially, the block (or ball) was resting on the edge of the opening so that it could be nudged in. After the birds nudged in the block on three consecutive trials, it was then placed on the surface in front of the apparatus so that subjects were required to pick it up and place it in the opening.

After three consecutive successes, the birds then moved to the final stage of training in which the tool and a non-functional object were presented equidistant from either side of apparatus (side of correct object counterbalanced across trials) and subjects were required to select the correct object and carry it to the apparatus for use. Once subjects made physical contact with one of the two objects, the experimenter approached from the other side and removed the remaining object. As the crows were less habituated to close human presence, the experimenter remained hidden outside of the testing compartment. If the bird attempted to approach the second object, the experimenter gave a soft knock on the door of the compartment, causing the crow to leave the testing table while the experimenter entered to remove the second object, after which the bird would come back down to the table to complete the task. This procedure had also been used for several weeks prior while training the birds to drop stones, and thus represented a minimal disturbance to the birds by the time of testing.

***Rigidity.*** Subjects were again trained in a step-wise procedure using the same criterion for progress as the weight set, beginning with the stick tool already inserted into the tube or crevice.

***Phase 3. Object exploration (post-training)***

Post-training exploration sessions followed the same protocol as Phase 1, with the exception that the baited apparatus corresponding to that set was now in view during exploration. For the kea, birds needed to walk past the apparatus in the testing compartment to enter the waiting compartment, and once in the waiting compartment the apparatus remained fully visible. For the crows, the apparatus was presented in the same compartment, but featured Perspex coverings to each of the openings so that birds could not use the objects on the apparatus in any way.

***Phase 4. Test trials***

During test trials the objects were paired randomly such that half of the trials featured objects from the same exploratory phase, whereas the other half featured one object from Phase 1 and one object from Phase 3. Between trials the experimenter re-baited the apparatus and placed new objects, all out of sight of subjects. If subjects did not make a choice within three minutes, or chose an object but did not carry it to the apparatus to retrieve the reward, a new trial was started and the incomplete trial was repeated at the end of the session.

### **Behavioural data**

All object exploration sessions (Phases 1 and 3) were coded in the Observer XT Version 10. A random selection of 12 videos (18%) was independently re-coded by a research assistant uninvolved in the study and blind to whether the session was pre- or post-tool use training, as well as which objects were functional versus non-functional. Interobserver reliability was substantial (Cohen’s kappa = 0.70) indicating the videos had been coded reliably.

Each interaction with the objects (when subjects were in physical contact with an object) was coded as a single bout, and each bout was coded hierarchically by assigning it to the behaviour category of whichever behaviour within the bout provided the most information about the structural properties of the object. For example, if a subject moved an object on the substrate before picking it up in the beak, this entire bout was coded as “pick up with beak”, whereas if the subject had only contacted the object with the beak, the bout was coded as “contact with beak” (see Table **S** for ethogram). Behaviours that potentially provided information about the structural properties of the objects, such as picking up a block, were further classified as ‘functional’ behaviours. Behaviours with less potential to gain information about structural properties, such as contacting a block with the beak, were classified as ‘non-functional’.

These behavioural data were then converted into several proportional measures for subsequent analyses. Both general exploratory behaviour (all behaviours in Table S2, including functional behaviours) and functional behaviours were examined as a proportion of the total trial time, either overall or separately for pre- and post-training sessions (Phase 1 and 3). Exploration of functional versus non-functional objects was measured as a proportion of the time the individual spent exploring the objects.

Table S2. Ethogram of behaviours coded for object exploration sessions, including which behaviours provided information about the structure of the different object sets (i.e., functional behaviours). Behaviours are listed in their hierarchical order.

| **Behaviour** | **Description** | **Functional?** |
| --- | --- | --- |
| *Throw* | Object is picked up from the substrate and momentum is used when releasing it from the beak, so that it travels some distance in the air rather than dropping straight from the beak. | Ropes, blocks |
| *Drag-throw* | The subject begins by dragging the object across the substrate with the beak, and the object breaks contact with the substrate while being dragged. | Ropes, blocks |
| *Pick up with beak* | Bird picks up the object with the beak so that it is no longer in contact with the surface it rests on (blocks) or only one end is in contact with surface (ropes) but does not throw or bring object into contact with any other surfaces or free objects. | Ropes, blocks |
| *Pick up with foot* | Bird picks up the object with the foot so that it is no longer in contact with the surface it rests on (blocks) or only one end is in contact with surface (ropes). | Ropes, blocks |
| *Insert* | Bird inserts the object through the fence or into a nook or crevice and completely lets go with the beak. | Ropes, blocks |
| *Probe* | Bird inserts the object through the fence or into a nook or crevice and retracts it entirely. | Ropes, blocks |
| *Drag* | Bird drags the object across the ground without the object leaving the substrate. | Ropes, blocks |
| *Move with beak* | Bird pushes object on substrate while contacting but not holding with the beak. Moves are classified as greater or less than 5cm. Moving was not considered a functional behaviour for the ropes as these objects usually retained their straight shape when moved. | Blocks (>5cm) |
| *Move with tool* | Bird uses a stick tool to move the object while maintaining contact between the tool and object for the duration of the behaviour. | Blocks (>5cm) |
| *Move with foot* | Bird pushes object on substrate while contacting with the foot. | Blocks (>5cm) |
| *Turn* | Specific to the block set: Kea turns the block over with the beak or foot so that it is now resting on a new side. |  |
| *Contact with beak and foot* | Bird contacts the object with both the beak and foot without moving the object on the substrate. |  |
| *Contact with beak* | Bird contacts the object with the beak (e.g. biting, touching with the outer part of the beak, etc.) without picking up the object or moving it on the substrate. |  |
| *Contact with foot* | Bird contacts the object with the foot but does not pick up or move on substrate. |  |

### **Supplementary Analysis**

#### Do birds alter their exploration to gain information about objects?

GLMMs 3-5 featured proportional data on birds’ exploration, which we transformed for normality using the arcsine squareroot transformation (Crawley 2011, p248). We note, however, that the arcsine transformation may not be a preferred method of data transformation (Warton et al. 2011), and we therefore ran additional pairwise comparisons corroborating the results of GLMMS 3-5. Wilcoxon signed rank tests found no significant difference between conditions for subjects’ average proportion of time spent exploring the objects (N = 14, *Z* = -.534, *p* = .636), engaging in functional behaviours (*Z* = -.534, *p* = .626), or interacting with functional relative to non-functional objects (*Z* = -.157, *p* = .903). Mann-Whitney U tests show that kea spent a significantly greater proportion of time interacting with the objects (*Z* = -2.840, *p* = .003), engaging in functional behaviours (*Z* = -2.582, *p* = .008) and exploring functional objects (*Z* = -2.324, *p* = .02).

**Results**

**Table S3.** Individual performance for test trials and exploration phases within each object set. Boxes show the number of objects of each type (functional or non-functional) contacted before the tool use task (grey) or after (diagonal stripes). For example, PI interacted with seven ropes (four functional, and three non-functional) before the training, and 10 ropes (5 of each type) after the training; JO interacted with 2 blocks (both functional) before the training and 2 blocks (one functional, one non-functional) after the training. Sub-adult individuals are *italicized.* Significantly above chance performance is marked with an asterisk*, and significantly below chance performance is marked with a ^Ϯ^.

**Table S4.** Performance of all subjects across trials for all experiments. Grey boxes (also marked with “1” denote a correct choice, while white boxes (also marked with a “0”) denote an incorrect choice. Total number of correct trials for each experiment and object set is shown in the “correct” column, with above chance performances in **bold**.

**References**

Bird, C. D. & Emery, N. J. (2009). Insightful problem solving and creative tool modification by captive nontool-using rooks. *Proceedings of the National Academy of Sciences* 106(25): 10370-10375.

Heinrich, B. (1988). Why do ravens fear their food? *Condor* 90: 950-952.

Honig, W. K. & Urcuioli, P. J. (1981). The Legacy of Guttman and Kalish (1956): 25 Years of Research on Stimulus Generalization) *Journal of the Experimental Analysis of Behavior* 36: 405-445.

Warton, D. I. & Hui, F. K. C. (2011). The arcsine is asinine: the analysis of proportions in ecology. *Ecology* 92(1): 3-10.
